# ProtoCell4P: an explainable prototype-based neural network for patient classification using single-cell RNA-seq

**DOI:** 10.1093/bioinformatics/btad493

**Published:** 2023-08-04

**Authors:** Guangzhi Xiong, Stefan Bekiranov, Aidong Zhang

**Affiliations:** Department of Computer Science, University of Virginia, Charlottesville, VA, United States; Department of Biochemistry and Molecular Genetics, University of Virginia, Charlottesville, VA, United States; Department of Computer Science, University of Virginia, Charlottesville, VA, United States

## Abstract

**Motivation:**

The rapid advance in single-cell RNA sequencing (scRNA-seq) technology over the past decade has provided a rich resource of gene expression profiles of single cells measured on patients, facilitating the study of many biological questions at the single-cell level. One intriguing research is to study the single cells which play critical roles in the phenotypes of patients, which has the potential to identify those cells and genes driving the disease phenotypes. To this end, deep learning models are expected to well encode the single-cell information and achieve precise prediction of patients’ phenotypes using scRNA-seq data. However, we are facing critical challenges in designing deep learning models for classifying patient samples due to (i) the samples collected in the same dataset contain a variable number of cells—some samples might only have hundreds of cells sequenced while others could have thousands of cells, and (ii) the number of samples available is typically small and the expression profile of each cell is noisy and extremely high-dimensional. Moreover, the black-box nature of existing deep learning models makes it difficult for the researchers to interpret the models and extract useful knowledge from them.

**Results:**

We propose a prototype-based and cell-informed model for patient phenotype classification, termed ProtoCell4P, that can alleviate problems of the sample scarcity and the diverse number of cells by leveraging the cell knowledge with representatives of cells (called prototypes), and precisely classify the patients by adaptively incorporating information from different cells. Moreover, this classification process can be explicitly interpreted by identifying the key cells for decision making and by further summarizing the knowledge of cell types to unravel the biological nature of the classification. Our approach is explainable at the single-cell resolution which can identify the key cells in each patient’s classification. The experimental results demonstrate that our proposed method can effectively deal with patient classifications using single-cell data and outperforms the existing approaches. Furthermore, our approach is able to uncover the association between cell types and biological classes of interest from a data-driven perspective.

**Availability and implementation:**

https://github.com/Teddy-XiongGZ/ProtoCell4P.

## 1 Introduction

As the basic unit of biological functions, the single cell is an ideal object to reveal personal characteristics and account for individuals’ differences in their phenotypes (e.g. race, disease state, etc.). Meanwhile, single-cell RNA sequencing (scRNA-seq) data possess powerful and high-resolution signatures which can be useful for precision medicine (He *et al.* 2021). Thus, it is tempting to use scRNA-seq data for the study of patients’ phenotypes, which may reveal the connection between patients’ phenotypes and gene expression data at the single-cell level and help researchers gain a deep understanding of the phenotype mechanisms.

**Figure 1.**
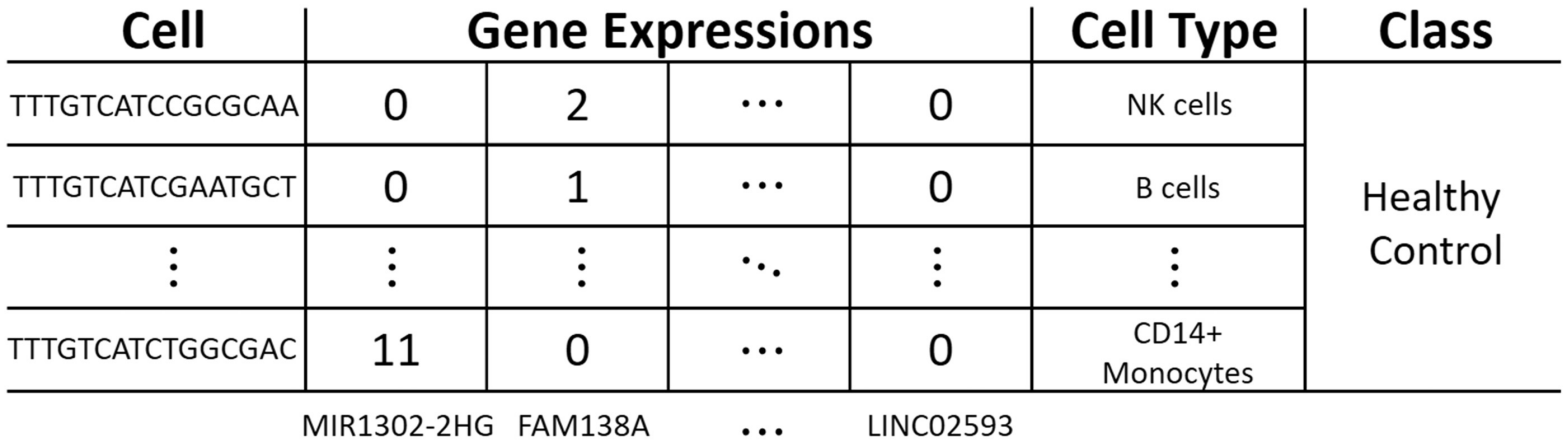
Illustration of one sample in a scRNA-seq dataset

Numerous studies have used scRNA-seq data to investigate the functions and types of different cells, revealing their interactions within humans and thereby providing an in-depth understanding of human health (Jovic *et al.* 2022). Powerful statistical and machine learning (ML) approaches have been developed for the analysis of scRNA-seq data, such as improving inference of gene expression (Breda *et al.* 2021), cell type identification (Huang and Zhang 2021), batch correction (Li *et al.* 2020), and clustering (Grønbech *et al.* 2020, Petegrosso *et al.* 2020). However, very little work has been done on the use of scRNA-seq data for the automatic classification of patients’ phenotypes, as the small number of patient samples in scRNA-seq datasets will prevent the classifiers from being well trained. Moreover, most of the classification methods cannot be applied since the samples collected in the same dataset contain a variable number of cells. In addition, the large amount of cells and high-dimensional gene expression data in an individual make modeling difficult and the predictions hard to explain, even if the classification results can be provided. Figure 1 illustrates the information contained by each sample (patient) in scRNA-seq datasets. For each sample, there is a class label that indicates the phenotype of the sample, such as healthy control. Also, each sample contains a set of cells and their cell types as well as the expressions of different genes in cells. In the same dataset, the genes selected for observation should be constant for different cells and different samples. However, the selection of cells may not be the same for samples, and even the number of cells recorded in them can be variable.

To tackle the aforementioned problems, we propose an interpretable **Proto**type-based and **Cell**-informed model for **P**atient classification tasks (termed **ProtoCell4P**) that leverages the knowledge of scRNA-seq data to predict individual phenotypes, where the prototypes are representatives of cells. The proposed model consists of a cell embedding module which encodes the cells into the latent space and learn a group of cell prototypes that can be representatives of cell subpopulations, and a classification module that adaptively evaluates the relevance of prototypes in the classification for each cell and combines the prototype-related information from all cells to make the final prediction. We propose to use cell-level encoders and relevance scorers to address the problem of few patient samples, as the large number of cells of each sample enables the model to fine tune the parameters of the cell-level neural networks even though the number of patient samples is small. Specifically, by pretraining the cell embedding module (Section 3.1) using a cell-level task, ProtoCell4P is able to capture concise and accurate cell information with the abundant single cells in scRNA-seq datasets, which allows the classification module to tune to the classifier well with a limited number of samples. The adaptive relevance estimation in the classification module (Section 3.2) will then help the model to fully leverage the classification knowledge contained in each cell by making cell-level predictions of the patients’ phenotypes. By averaging the cell-level predictions as the final results, ProtoCell4P is capable of processing a variable number of cells and can achieve accurate classification for the patient samples.

Beyond patient classifications, our design of the model makes it able to identify the key cells that play a crucial role in the classification task, and we also incorporate the fusion of cell type knowledge into the model during training to enhance the model’s interpretability from the perspective of cell types. By regularizing the latent space to be cell-type informative and encouraging the model to provide cell-type specific relevance estimation for cells, we are able to link the classification of unknown phenotypes of patients to our existing knowledge of cell types, thus uncovering the associations between them, which can facilitate both clinical diagnosis and biological research. Our contributions can be summarized as follow:

1. We propose a new approach to utilize scRNA-seq data for patient classifications, which overcomes the problem of sample scarcity by fully leveraging the knowledge of each cell.
2. Our approach is explainable at the single-cell resolution which can identify the key cells in each patient’s classification.
3. We generalize the information of cells with their cell type information, and make the model able to provide higher-level interpretation of its effective reasoning process.
4. Our model can be used for the discovery of unknown associations between cell types and phenotypes, which can be enlightening for biological research.

## 2 Related work

*ML on scRNA-seq data.* Since the invention of scRNA-seq technology, many ML-based approaches have been proposed for single-cell analysis. Research questions such as imputation (Xu *et al.* 2020), batch effect correction (Johnson *et al.* 2019), and cell-type identification (Lopez *et al.* 2018) based on scRNA-seq data have received a lot of attention, and a large number of ML approaches have been implemented on these scRNA-seq based problems. However, these approaches only focus on the study of single cells and do not address the patient classification from whom the cells are derived, likely because of limited patient sample size and it is difficult to determine what roles different cells play when classifying individuals into different classes.

CloudPred (He *et al.* 2021) was proposed to predict patient phenotypes based on scRNA-seq data using a Gaussian Mixture Model, which encodes each cell and takes their average as the feature for the individual. This approach, however, does not consider the cell differences in their relevance to the classification task, and it is hard to identify the contribution of each cell since this information is entangled in CloudPred’s quadratic classifier. In contrast, our method can adaptively estimate the importance of each cell in the classification, which can increase both the performance of the model and the interpretability of each cell’s role in the classification.

*Prototype-based neural network.* Prototype-based classification is a classic way to perform case-based reasoning (Kolodner 1992), which learns to classify a given input by comparing it to the typical examples that the system has seen before. There are already some works that tried to incorporate prototypes in ML models for interpretable classification (Bien and Tibshirani 2011, Kim *et al.* 2014). Using the autoencoder to encode and decode image data, Li *et al.* (2018) proposed to learn prototypes in the latent space and classify the input image based on its distances to different prototypes in the latent space, thus making their DNN model interpretable by looking at the model’s learned weights on different prototypes for the classification task. Following up on this work and the idea from Alvarez Melis and Jaakkola (2018) and Huai *et al.* (2022) proposed to learn the weights on the prototypes dynamically for different inputs, which they called importance scores. However, a big issue with these approaches is that the interpretation of their models’ effective reasoning processes remains unclear with the distances to prototypes as classification features, since the small distance and the high weight will obscure the contribution of a prototype in the final prediction. In ProtoCell4P, we propose to calculate the similarity between the input and the prototype instead of the distance, which avoids the problem in the above methods. Our preliminary experiments also show that if we use their distance metrics instead of our similarity, it is always those points distant from the prototypes that have a significant impact on the classification.

Prototype-based approaches are normally applied to tasks on image data, since the prototypes of images can be conveniently visualized with the decoder to see what has been learned by the model. However, single-cell data are not nearly as human interpretable as image data. Instead of trying to decode the latent prototypes, Brbić *et al.* (2020) visualized what the model has learned in the latent space by reducing the dimension of the learned embeddings and plotting them on a 2D figure using t-SNE (Van der Maaten and Hinton 2008) and UMAP (McInnes *et al.* 2018). In their work, they let the model learn prototypes of cells in order to predict cell types for cells from different datasets. Cao *et al.* (2021) also implemented their prototype-based approach on single-cell data by making the model learn prototypes of gene concepts and perform few-shot cell type classification on that basis. These approaches have demonstrated that it is feasible to learn prototypes of cells and use them in downstream tasks. However, they are not applicable to our patient classification task since they only considered tasks on single cells just like other ML approaches for the scRNA-seq data.

## 3 Methodology

**Figure 2.**
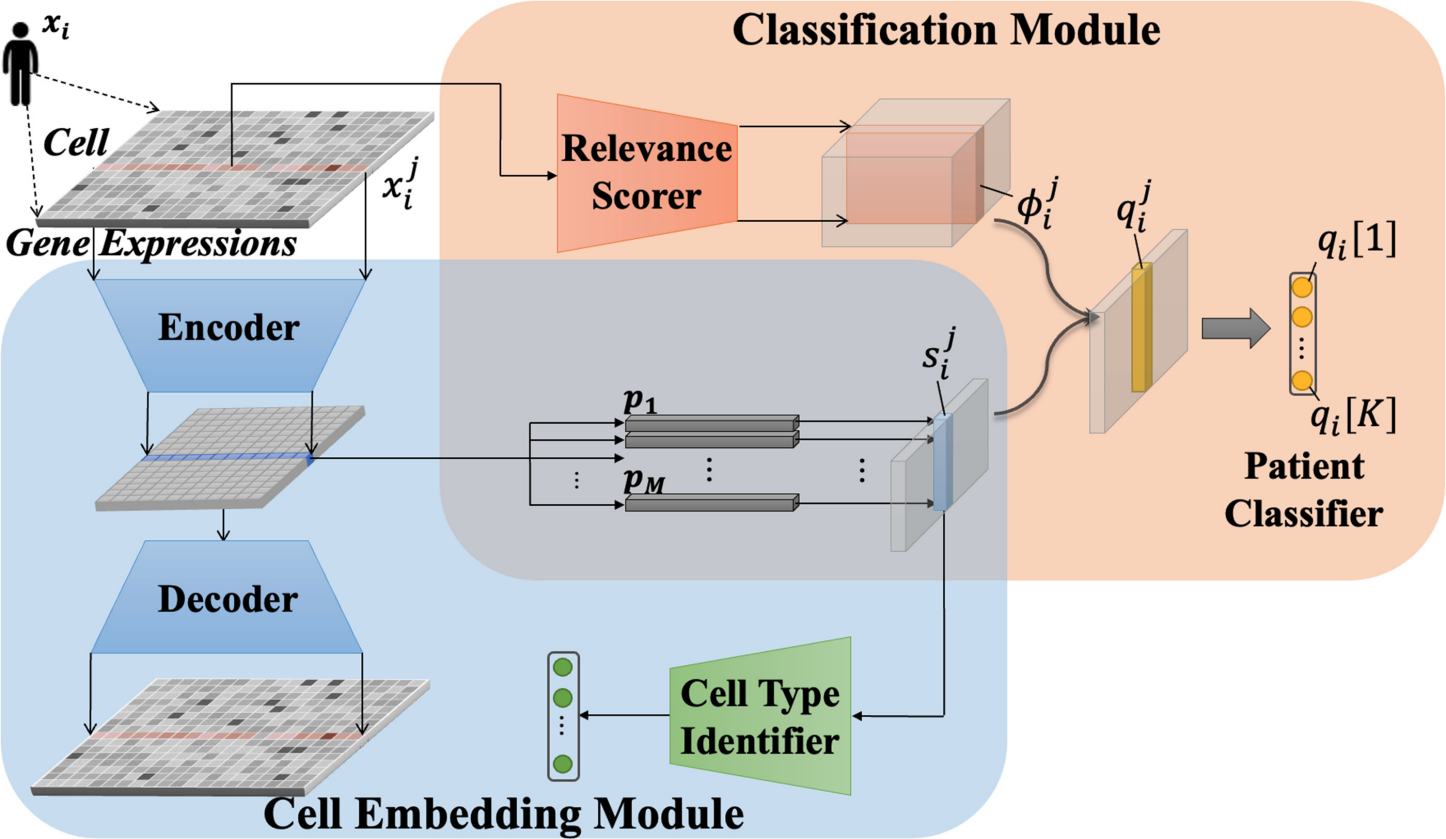
Architecture of ProtoCell4P

Our model for patient classification, ProtoCell4P, consists of two components. First, it has a cell embedding module, which encodes the input gene expression data of a single cell into a low-dimensional latent space and learns embedded cell prototypes automatically. Second, it has a classification module, which classifies an individual by estimating dynamic relevance scores of prototypes for each cell and integrating the information from all cells in a sample. The overall architecture of ProtoCell4P is shown in Fig. 2.

### 3.1 The cell embedding module

*Cell and prototype embeddings.* As the architecture shows, the autoencoder structure (Ballard 1987) is used to encode the information of cells into the latent space. Given the genetic profile of an input cell, the encoder *f* will return a latent embedding that will then be received by the decoder *g* to recover the original input. In order to encourage the encoder to fully extract useful information from the cell data, the reconstruction loss term is penalized during the training of ProtoCell4P. For a batch input with *m* samples, the reconstruction loss is defined as where $x_{i}^{j}$ stands for the gene expression data for the *j*th cell of the *i*th sample in the batch and $n_{i}$ is the number of cells contained in the data of the *i*th sample. *N* is the total number of cells in the batch, which is computed by $N=\sum_{i=1}^{m}n_{i}$.


(1) $$L_{\text{recon}}=\frac{1}{N}\sum_{i=1}^{m}\sum_{j=1}^{n_{i}}\left|\right|x_{i}^{j}-g(f(x_{i}^{j}))|{|}_{2}^{2},$$

To increase the model’s interpretability in patient classification, we adopt the concept of prototypes and introduce cell-prototypes for patient classification, which are considered to be representatives of cell subpopulations. In our implementation, the prototypes are initiated by the model as random vectors, which are then updated according to the training objectives using gradient descent.

To encourage the cells to cluster around the prototypes, we add a constraint to the latent space that promotes the embedding of a cell close to at least one cell-prototype. This constraint can be formalized as the minimization of the cell-to-prototype distances where *M* is the number of cell-prototypes in ProtoCell4P and $p_{1},\ldots ,p_{M}$ are the cell-prototypes learned by the model.


(2) $$L_{c2p}=\frac{1}{N}\sum_{i=1}^{m}\sum_{j=1}^{n_{i}}\underset{k\in [1,M]}{\min}||f(x_{i}^{j})-p_{k}|{|}_{2}^{2},$$

In addition to shortening the distance between cells and cell-prototypes to learn meaningful subpopulation representatives, we also maximize the prototype-to-prototype distance to disperse the cell-prototypes and thus distinguish different cell subpopulations in the latent space. The loss term for the prototype-to-prototype distance penalty is formulated as where $d_{\text{min}}$ is the minimum acceptable pairwise distance between two cell-prototypes.


(3) $$L_{p2p}=\frac{2}{M(M-1)}\sum_{k=1}^{M-1}\sum_{l=k+1}^{M}\max{\left(0,d_{\text{min}}-||p_{k}-p_{l}|{|}_{2}\right)}^{2},$$

*Cell type knowledge fusion.* Since the cell types represent the existing knowledge of cells, the latent space will be interpretable if the learned cell subpopulations can be related to the cell types that people have already known. As the cell type labels are easily accessible from human-annotated datasets or automatic annotation tools (Ianevski *et al.* 2022), we take them as the given information in the scRNA-seq datasets and include them as parts of the training data. To encourage the model to learn cells of the same type as cell subpopulations and take the cell-prototypes as the representatives, we propose to add constraints on the distance from prototypes to groups of cells of the same type. Specifically, for cells with the same cell type label, we take the average of their cell embeddings as the latent representation of that cell type. By penalizing the distance from each cell-prototype to the closest cell type embedding, we can encourage the cell-prototype to learn the knowledge of cell types and be the corresponding representative. The penalty term can be formulated as where *T* is the total number of cell types and $1_{t}(x_{i}^{j})$ is an indication function defined as


(4) $$\frac{1}{M}\sum_{k=1}^{M}\underset{t\in [1,T]}{\min}{\left\|p_{k}-\frac{\sum_{i=1}^{m}\sum_{j=1}^{n_{i}}1_{t}(x_{i}^{j})f(x_{i}^{j})}{\sum_{i=1}^{m}\sum_{j=1}^{n_{i}}1_{t}(x_{i}^{j})}\right\|}_{2}^{2},$$

(5) $$1_{t}(x_{i}^{j})=\left\{\begin{array}{c} 1\;\text{if cell}\;x_{i}^{j}\;\text{is of the}\;t\text{th type},\\ 0\;\text{if cell}\;x_{i}^{j}\;\text{is not of the}\;t\text{th type}. \end{array}\right.$$

In our preliminary experiments, we found that the model sometimes assigned more than one cell-prototype to the same cell type, and they would overlap each other when we visualized the latent space on a 2D figure. To diversify the cell types associated with the learned cell-prototypes and to make the prototype learning process robust, we propose to add randomness in determining the closest cell type population for each prototype in Formula (6). Specifically, the distance from a cell-prototype to a cell type embedding will have the probability of a large value being added, which prevents the cell-prototype from always binding to a specific cell type, thus increasing the chance that these cell-prototypes have diverse related cell types. In our experiments, the loss term in Formula (4) is updated as where and β is set as 0.3 in our experiments.


(6) $$\begin{array}{c} L_{p2\text{ct}}=\frac{1}{M}\sum_{k=1}^{M}\underset{t\in [1,T]}{\min}\{10^{9}\delta_{tk}+\\ {\left\|p_{k}-\frac{\sum_{i=1}^{m}\sum_{j=1}^{n_{i}}1_{t}(x_{i}^{j})f(x_{i}^{j})}{\sum_{i=1}^{m}\sum_{j=1}^{n_{i}}1_{t}(x_{i}^{j})}\right\|}_{2}^{2}\}, \end{array}$$

(7) $$\delta_{tk}∼\text{Bernoulli}(\beta ),$$

Additionally, we need to encourage the cells of the same type to be close to each other, because even under all of the above constraints, the type of cells surrounding the learned prototype is not necessarily the same as the type represented by the prototype. We realize this by adding a cell type identifier to the module, which takes the information on the similarity of a cell to each cell-prototype as the input feature. To be specific, we can obtain a cell’s similarity vector by first computing its distance to different cell-prototypes in the latent space and then transforming it into the inverse to describe the cell’s similarity to different subpopulations where ϵ=0.5 in our experiments. We introduce a linear classifier *F* to make predictions about cell types based on the similarity vector calculated in the previous step. The linear classifier is chosen because we expect that the cell-prototypes will contain enough information to enable the basic classifier to make accurate cell type predictions. Training of the cell type identifier will in turn prompt the model to learn critical cell-prototypes that can be used to differentiate different types of cells according to their locations in the latent space. The loss for the cell type identification is where $F(s_{i}^{j})[t]$ stands for the *t*th element of the output prediction $F(s_{i}^{j})$.


(8) $$s_{i}^{j}={\left[\frac{1}{||f(x_{i}^{j})-p_{1}|{|}_{2}^{2}+\epsilon },\ldots ,\frac{1}{||f(x_{i}^{j})-p_{M}|{|}_{2}^{2}+\epsilon }\right]}^{⊤},$$

(9) $$L_{\text{ct}1}=-\frac{1}{N}\sum_{i=1}^{m}\sum_{j=1}^{n_{i}}\sum_{t=1}^{T}1_{t}(x_{i}^{j})\;\text{log}\;F(s_{i}^{j})[t],$$

### 3.2 The classification module

In the above subsection, we described how to compute the similarity vector of a cell over all cell-prototypes to classify its cell type. However, such an approach cannot be directly applied to our patient classification of interest, since numerous cells can be included within one individual. To precisely predict the phenotype of an individual, we need to integrate information from all its cells to make a final prediction. We propose a cell-informed patient classification method, which combines the well-trained embeddings of all cells using an adaptive relevance scorer to effectively retrieve useful information from the cells for the classification task.

*Adaptive relevance scorer.* It is crucial to estimate the relevance of each cell-prototype for patient classification since the cell-prototypes may have different effects and should be given different weights in the classification task. Moreover, it is also necessary to take into account the possible differences between individuals and between cells. According to Perez *et al.* (2022), in some scenarios, not all types of cells have significantly different behaviors in cohorts with different phenotypes. To cope with these situations, we propose an adaptive relevance scorer to control the contribution of each cell to the final prediction, thus personalizing the precise diagnosis for each patient.

A neural network Φ is proposed as the relevance scorer, which aims to output an estimated relevance score matrix for each cell to show the relevance of that cell’s similarity to each cell-prototype for classifying the patient’s phenotype. In other words, the relevance scores will indicate how much the similarities between the cell and the cell-prototypes contribute to categorizing the individual into each target class. Given the data of $n_{i}$ cells from a patient, the scorer Φ will return $n_{i}$ different importance matrices, each of size M×K, where *M* and *K* are the numbers of cell-prototypes and target classes, respectively.

Combining the computed similarity vector (Formula (8)) and the estimated relevance score matrix given by $\Phi (x_{i}^{j})$, ProtoCell4P can calculate the importance scores of a cell $x_{i}^{j}$ in identifying the individual $x_{i}$’s phenotype via


(10) $$q_{i}^{j}=s_{i}^{j⊤}\Phi (x_{i}^{j}).$$

By summing up the results from each cell, ProtoCell4P can give a final estimation of the classification probability as


(11) $$q_{i}=\text{Softmax}(\sum_{j=1}^{n_{i}}q_{i}^{j}).$$

The error for the patient classification is defined as the cross entropy between the true labels and the estimated label distributions, defined as where $q_{i}[r]$ is the *r*th element of the prediction $q_{i}$ for the *i*th individual, and $1_{r}^{\prime }(x_{i})$ is an indication function defined as


(12) $$L_{\text{clf}}=-\frac{1}{m}\sum_{i=1}^{m}\sum_{r=1}^{K}1_{r}^{\prime }(x_{i})\;\text{log}\;q_{i}[r],$$

(13) $$1_{r}^{\prime }(x_{i})=\left\{\begin{array}{c} 1\;\text{if sample}\;x_{i}\;\text{is of the}\;r\text{th class},\\ 0\;\text{if sample}\;x_{i}\;\text{is not of the}\;r\text{th class}. \end{array}\right.$$

Besides, to improve the interpretability of ProtoCell4P in terms of cell types, we encourage the estimated relevance score matrices for the same type of cells to have similar patterns so that, given their prototype-related information should be close based on our design of the cell embedding module, these cells will have similar contributions to the classification, which helps to explore the association between phenotype classes and cell types. To be specific, we introduce a new cell type classifier in the classification module, which takes the estimated relevance scores as the input. Different from the cell type identifier in the cell embedding module, this classifier does not predict the cell types based on the embeddings but on the relevance only, in order to regulate the behavior of the same type of cells in the patient classification task. Noting the cell type classifier as G, we need to penalize the following classification error where *T* is the number of cell types in total and $G(\Phi (x_{i}^{j}))[t]$ denotes the *t*th element of the prediction $G(\Phi (x_{i}^{j}))$.


(14) $$L_{\text{ct}2}=-\frac{1}{N}\sum_{i=1}^{m}\sum_{j=1}^{n_{i}}\sum_{t=1}^{T}1_{t}(x_{i}^{j})\;\text{log}\;G(\Phi (x_{i}^{j}))[t],$$

### 3.3 Training procedure and interpretability

*Overall training procedure.* The overall model is trained with a two-step approach using the same training set. First, we pretrain the cell embedding module in ProtoCell4P to learn explicit and identifiable cell embeddings and cell-prototypes in the latent space. The well-encoded cell embeddings and prototypes will then facilitate the second module to quickly learn how to classify patients in the face of sample scarcity.

In the pretraining phase, sequencing data and cell type labels of single cells from patient samples in the training set are used to train the cell embedding module with the cell type classification as the task (Formula (9)). Meanwhile, we include the optimization of the reconstruction loss (Formula (1)) and the distance-based penalty of embeddings (Formulae (2), (3), and (6)) to encourage the learning of informative cell embeddings and prototypes. The training objective of the model in this phase should be the combination of all loss terms mentioned in Section 3.1 where $\lambda_{1},\ldots ,\lambda_{5}$ are the weights we put on different loss terms, guiding the model to give different attention to the optimization objectives.


(15) $$L_{1}=\lambda_{1}L_{\text{recon}}+\lambda_{2}L_{c2p}+\lambda_{3}L_{p2p}+\lambda_{4}L_{p2\text{ct}}+\lambda_{5}L_{\text{ct}1},$$

The optimal parameters of the cell encoder which provide the best cell type classification accuracy on the validation set will be kept for further training. In the tuning of the patient classification module, we fix the learned parameters in the previous phase and update the relevance scorer for better patient classification performance. Specifically, the model optimizes the patient classification objective with the scRNA-seq data and the phenotype labels of patient samples (Formula (12)), and learns to identify cell type information from the estimated relevance scores (Formula (14)) to encourage homogeneity of prediction patterns of cells of the same type. The objective of the second training stage is where $\lambda_{6}$ is the hyperparameter for the cell type classification loss in the training of the relevance scorer Φ.


(16) $$L_{2}=L_{\text{clf}}+\lambda_{6}L_{\text{ct}2},$$

*Interpretability of patient classification.* The estimated relevance score matrix can be used to show the role each cell plays in determining the phenotype class of the patient. If the relevance score of one prototype is high for a target class according to a given single cell, then the closer the cell is to the prototype, the more likely the patient will be assigned to the corresponding class. Moreover, by combining the similarity information on prototypes and corresponding relevance scores, we can obtain the importance of each cell which shows how much influence a cell has on the categorization of the sample into each phenotype. It is worth noting that if a cell is important for all phenotypes, then it will make no contribution to the final classification. By calculating the difference between the importance score of a cell for one category and its score for other categories, we can quantify how much the cell contributes to classifying the patient into that specific class. The contribution that the *j*th cell of the *i*th sample makes to classifying the patient into the κth class can be formulated as where $q_{i}^{j}[r]$ stands for the *r*th element of $q_{i}^{j}$ which is defined in Formula (10). Comparing the contribution scores of different cells, we can figure out which cells or cell subpopulations are more important for the patient classification tasks.


(17) $$c_{i}^{j}[\kappa ]=q_{i}^{j}[\kappa ]-\frac{1}{K-1}\left(\sum_{r=1}^{K}q_{i}^{j}[r]-q_{i}^{j}[\kappa ]\right)$$

## 4 Experiment

### 4.1 Datasets

We have chosen the lupus dataset (Mandric *et al.* 2020) since the method we compare with, CloudPred (He *et al.* 2021), used this dataset. The cardio (Chaffin *et al.* 2022) dataset and the covid dataset (Ziegler *et al.* 2021) are also used in our study. Table 1 provides a summary of real-world datasets that we use to evaluate ProtoCell4P on patient classification tasks.

**Table 1.** Datasets for the evaluation of patient classification (numbers in brackets indicate the distribution of labels).

Dataset name	No. of samples	Avg. no. of cells per sample	No. of cell types	No. of genes	No. of classes
Lupus (disease) (Mandric *et al.* 2020)	169 (119 + 50)	4935	8	32 738	2
Lupus (race) (Mandric *et al.* 2020)	169 (106 + 63)	4935	8	32 738	2
Cardio (Chaffin *et al.* 2022)	42 (11 + 15 + 16)	14 111	13	36 601	3
Covid (Ziegler *et al.* 2021)	58 (35 + 23)	562	18	32 871	2

### 4.2 Experimental setup

*Baseline approaches.* Due to its novel and complex nature, classifying patients based on single-cell information has been poorly explored. To the best of our knowledge, CloudPred (He *et al.* 2021) is currently the only approach that performs the single-cell based patient classification task using ML models. We apply CloudPred to all tasks we designed and compare its performance of patient classification with our model. In addition, to evaluate how much adaptive relevance scores will contribute to patient classification, we design another neural network based model, termed BaseModel. Following Li *et al.* (2018)’s approach, BaseModel uses prototypes to obtain the feature of each cell and uses a fully connected layer to provide probability estimation based on this. The way we train the model and combine the information from different cells in ProtoCell4P is also applied to the training of BaseModel and its classification of patients. The main difference between these two approaches is that BaseModel always gives a static matrix of relevance scores, rather than a dynamic one as in ProtoCell4P. In addition to CloudPred and BaseModel, we also test the baseline models that CloudPred used on our designed tasks, the descriptions of which can be found in the original paper (He *et al.* 2021).

### 4.3 Evaluation of performance

*Comparison with baselines.*  Tables 2 and 3 show the results of performance comparisons under different evaluation metrics, where the top three scores for each dataset are in bold. The results demonstrate that ProtoCell4P can effectively utilize single-cell information to identify the phenotype difference among patients, as evidenced by ROCAUC scores consistently above 0.90. With the ROCAUC score as the evaluation metric, our model outperforms CloudPred and other baseline models on all tasks.

**Table 2.** Comparison of performance with baselines (ROCAUC).

Method	No. of prototypes	Lupus (disease)	Lupus (race)	Cardio	Covid
Independent	–	0.98	0.80	0.64	0.86
Mixture (class)	–	0.99	0.88	0.85	**0.89**
Mixture (patient)	–	0.93	0.73	0.77	0.68
Deepset	–	1.00	0.89	0.67	**0.86**
CloudPred	–	1.00	0.90	0.86	0.81
BaseModel	–	0.88	0.67	0.68	0.64

ProtoCell4P	4	**1.00**	**0.98**	**0.93**	0.86
	8	**1.00**	**0.97**	**0.95**	0.86
	16	**1.00**	**0.97**	**0.96**	**0.90**

While the ROCAUC score measures the area under the receiver operator characteristic curve (ROC) and takes the predicted probabilities as the input, the F1 score requires the definition of probability thresholds of classes to obtain the predicted classes and then measures the harmonic mean of the precision and recall. Generally, a model with a dominant curve in the ROC space should also have a dominant curve in the precision–recall (PR) space (Davis and Goadrich 2006), and the computed F1 score corresponds to a specific point on the PR curve. As can be seen from the tables, though our model still has state-of-the-art performance in terms of the macro F1 score, the results are less exceptional with macro F1 scores <0.90 on the cardio and covid datasets. These results show that while ProtoCell4P is able to differentiate phenotypic differences among samples using the single-cell information, they may not always give the accurate label prediction of the patient phenotypes, especially on small-size datasets such as cardio and covid. The probability boundary of classes should be more carefully determined when implementing ProtoCell4P on these datasets.

**Table 3.** Comparison of performance with baselines (macro F1).

Method	No. of prototypes	Lupus (disease)	Lupus (race)	Cardio	Covid
Independent	–	0.89	0.65	0.40	0.41
Mixture (class)	–	**0.99**	0.78	**0.80**	**0.73**
Mixture (patient)	–	0.89	0.71	0.69	0.63
Deepset	–	0.99	0.80	0.20	0.41
CloudPred	–	0.97	0.79	0.65	**0.72**
BaseModel	–	0.70	0.47	0.28	0.36

ProtoCell4P	4	0.94	**0.86**	0.79	**0.72**
	8	**1.00**	**0.91**	**0.79**	0.62
	16	**1.00**	**0.90**	**0.82**	0.69

Comparing ProtoCell4P with BaseModel, we see that the adaptive relevance scorer does play a very significant role in patient classification and dramatically increases the performance of our model on the tasks. We also find that the optimal number of prototypes for ProtoCell4P appears to be different across tasks.

*Analysis of interpretability*. In addition to the good performance of our model on patient classification tasks, ProtoCell4P is also capable of showing its effective reasoning process for classification. The relevance scores can tell us, for each cell, the similarity to which prototype is more emphasized in the classification. By combining the information from various prototypes and relevance scores, we can identify the cells and cell types that are most important for classification, thereby uncovering the association between cells and target phenotype classes. Here we use case studies of the classification tasks to show the interpretation that ProtoCell4P can provide for its classification results.

For each task, we use t-SNE to visualize the cell embeddings and prototypes in the latent space. In the visualization results, cells of different types are reflected by different colors of the points, and we use pentagons (black) to show where the learned prototypes are located in the latent space. To visualize the position of the most important cells for the patient being classified, we first rank all cells according to their contributions using Formula (17). Then for each target class, we select the top 20 most important cells and plot them on the figure with the “+” signs.

**Figure 3.**
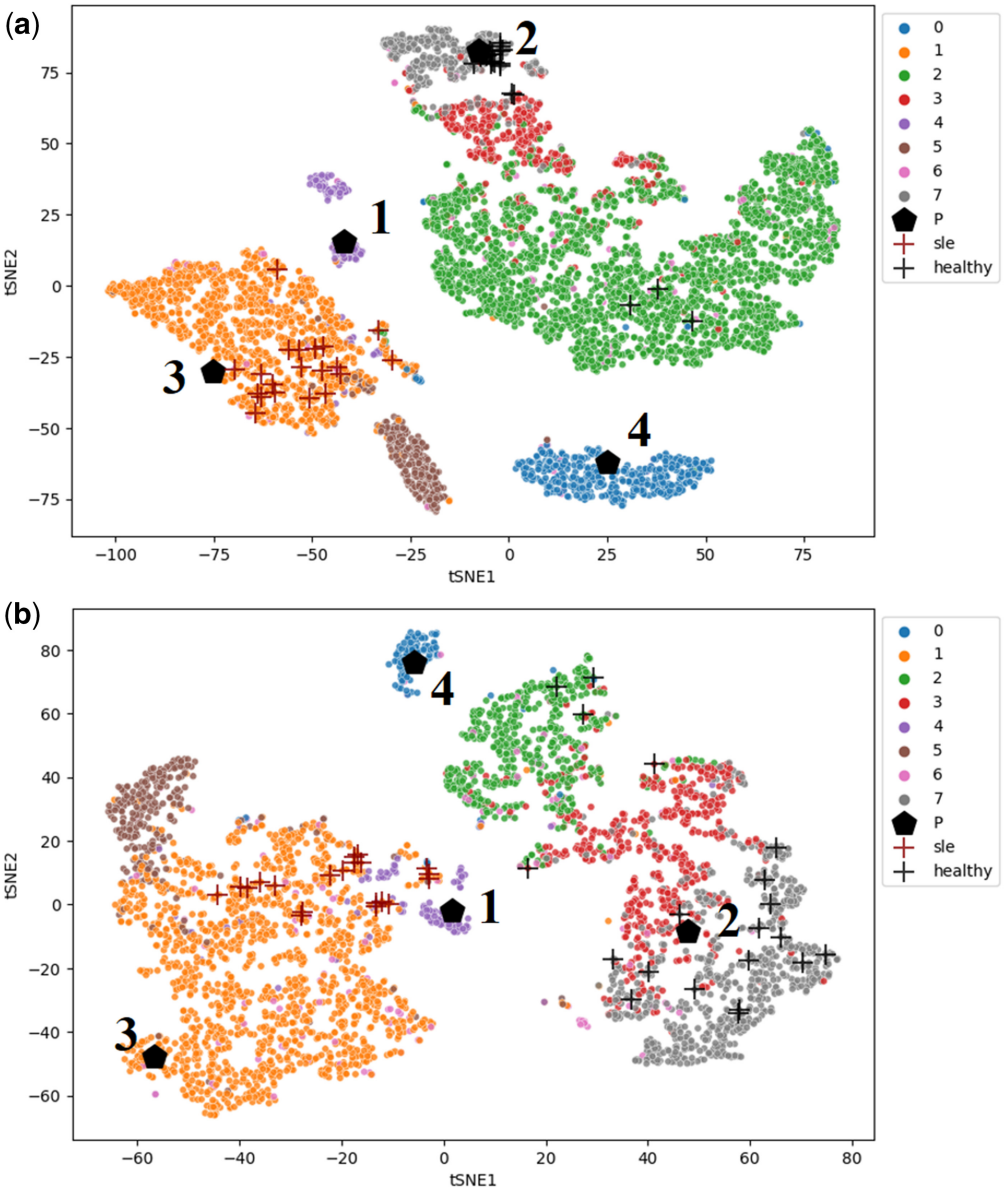
Latent space of cells with (a) a healthy sample and (b) an SLE sample in lupus

*Case study of lupus classification.* According to Fig. 3, embeddings of the same type of cells in lupus tend to gather as a cluster, and the prototypes are automatically learned to represent different cell types. For example, from Fig. 3a and b we can see that Prototypes 1, 2, 3, and 4 represent dendritic cells (purple), NK cell (gray), CD14+ Monocytes (orange), and B cells (blue), respectively. Though both CD8 T cells (red) and NK cell (gray) are close to Prototype 2, their estimated relevance scores show that the similarity to the prototype has a different impact on the contribution of these two cell types to the classification task. For the CD8 T cell subpopulation, the mean relevance score of its cell is 0.19 to the healthy class and 0.72 to the SLE class, while for the NK cell subpopulation, they are 0.73 and 0.80, respectively. In other words, CD8 T cells that are close to Prototype 2 will make the patient more likely to be classified as SLE, while the distance from NK cells to the prototype will not contribute much to differentiating two target classes for the patient.

From Fig. 3b for the SLE sample, it is shown that the most important cells for classifying the patient belonging to the SLE group are mainly from the CD14+ Monocyte subpopulation (orange), while cells that make the most contribution to pushing it to the healthy group are from the NK cell (gray) and CD4 T cell (green) subpopulations. Such patterns are also shown in Fig. 3a for the healthy sample. Comparing the distribution of cell types in both samples, we find that the SLE sample does have more CD14+ Monocytes (orange) and fewer CD4 T cells (green), which may account for its classification result as SLE. By averaging the contribution scores of all cells in each subpopulation, we obtain Table 4, which explicitly shows the association between cell types and different phenotype classes in the lupus classification task. In particular, we mark in bold the cell types that contributed the most to different phenotypes. The table shows that CD4 T cells and NK cells are positive signals for individuals to be classified in the healthy group, while CD14+ Monocytes and FCGR3A+ Monocytes will increase the likelihood of patients being classified as SLE. These are consistent with the findings of previous biological research (Sibbitt *et al.* 1983, Henriques *et al.* 2013, Perez *et al.* 2022, Zhang and Lee 2022).

**Table 4.** Average contribution scores of each cell type to the lupus classification.

	B cells	CD14+ monocytes	CD4 T cells	CD8 T cells	Dendritic cells	FCGR3A+ Monocytes	Megakaryocytes	NK cells
Healthy	−0.64	−3.89	**0.44**	−1.65	−1.47	−2.32	−2.15	**0.01**
SLE	0.64	**3.89**	−0.44	1.65	1.47	**2.32**	2.15	−0.01

**Figure 4.**
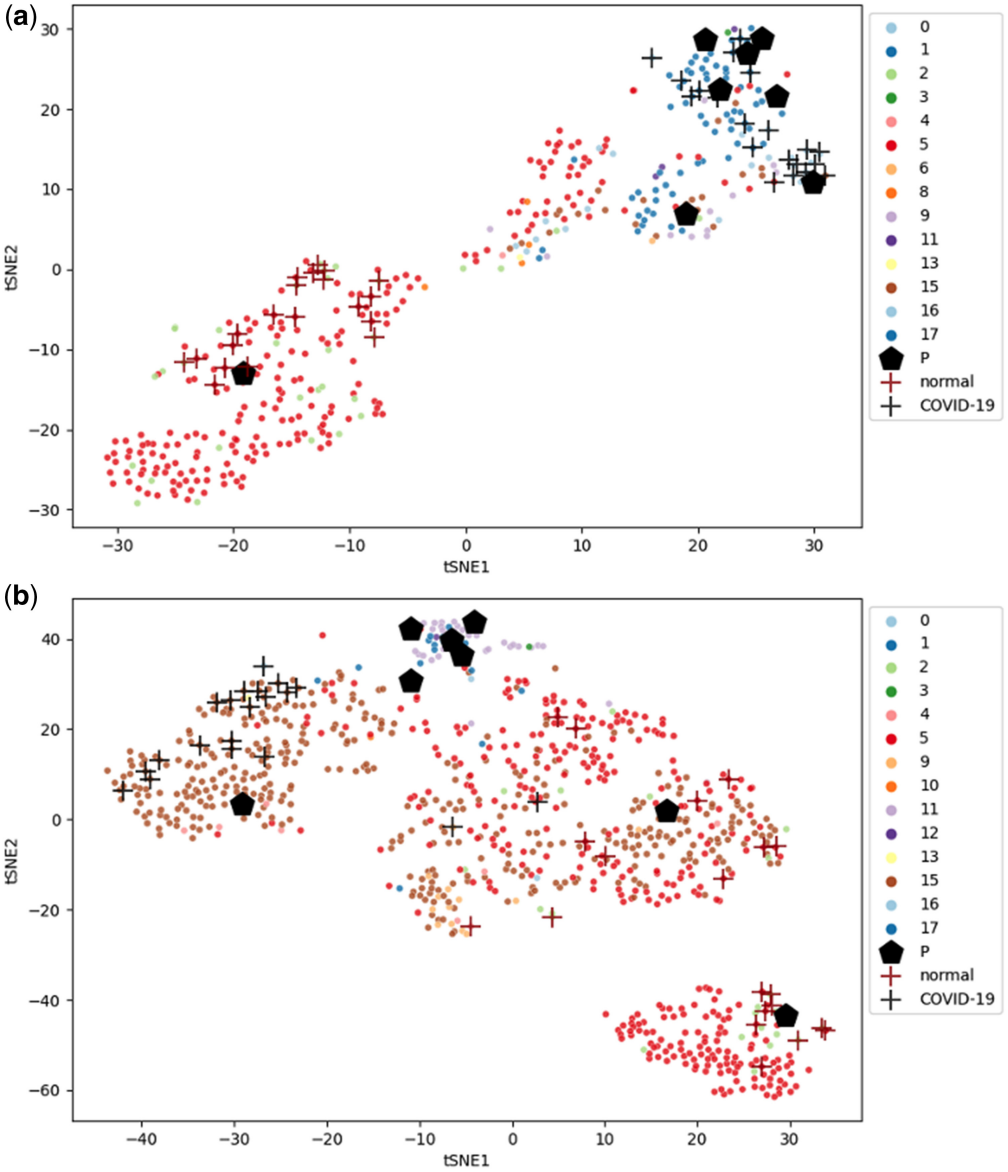
Latent space of cells with (a) a normal sample and (b) a covid sample in covid dataset

*Case study of covid classification*. Figure 4 and Table 5 show our experimental results on the covid dataset, with the cell types that contribute most to the classification of covid disease highlighted in Table 5. According to Table 5, our model identifies mast cells, goblet cells, and secretory cells as important indicators of the covid disease. The visualization results of cases (Fig. 4) show that some developing ciliated cells (red) and secretory cells (brown) play important roles in the classification of the patients’ covid disease state. These cell types are consistent with the recent research interest in COVID-19 (Chua *et al.* 2020, Ahn *et al.* 2021, Wu *et al.* 2021).

**Table 5.** Average contribution scores of each cell type to the covid classification.

	B cells	Basal cells	Ciliated cells	Dendritic cells	Deuterosomal cells	Developing ciliated cells	Developing secretory and goblet cells	Enteroendocrine cells	Erythroblasts
Normal	−1.49	−1.20	0.83	−1.45	−0.95	−0.55	−0.35	−0.78	0.08
Covid	1.49	1.20	−0.83	1.45	0.95	0.55	0.35	0.78	−0.08

	**Goblet cells**	**Ionocytes**	**Macrophages**	**Mast cells**	**Mitotic basal cells**	**Plasmacytoid DCs**	**Secretory cells**	**Squamous cells**	**T cells**

Normal	−2.20	−1.67	−1.20	−3.25	−1.27	−1.07	−2.60	−1.40	−1.25
Covid	**2.20**	1.67	1.20	**3.25**	1.27	1.07	**2.60**	1.40	1.25

### 4.4 Ablation studies

To fully understand how different components in ProtoCell4P contribute to the model’s performance and interpretability, we conduct ablation studies for ProtoCell4P on all datasets. We test the performance of ProtoCell4P in the absence of each component. The evaluation results are shown in Table 6. By removing the pretraining component, the model does not adopt the two-step training strategy as described in Section 3.3. Instead, it trains the entire model end-to-end, optimizing all objectives simultaneously. In experiments without cell type information, the model does not take the cell type annotations as its input and discards all training objectives regarding cell types in its training. We also test the model’s performance when the subsampling is not implemented as described in Section 4.2.

**Table 6.** Ablation studies for ProtoCell4P (ROCAUC).

Component	Lupus (disease)	Lupus (race)	Cardio	Covid
ProtoCell4P	1.00	0.98	0.96	0.90
Pretraining	1.00	0.96	0.94	0.90
Cell type	1.00	0.98	0.98	0.89
Subsample	1.00	0.97	0.96	0.92

**Figure 5.**
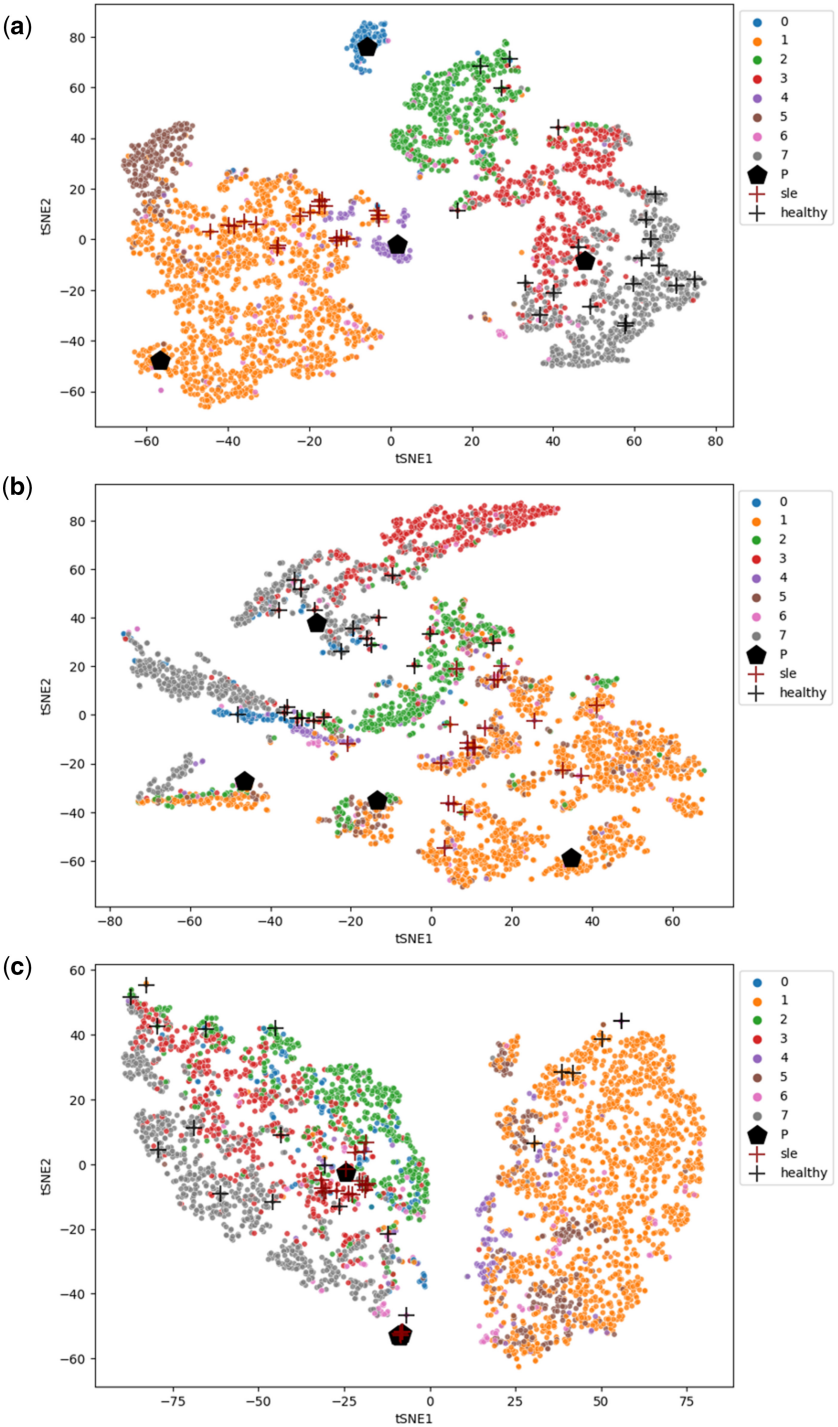
Cell embeddings of the same SLE sample learned by (a) ProtoCell4P, (b) ProtoCell4P without pretraining, and (c) ProtoCell4P without cell type

From the results, we can notice that the performance of ProtoCell4P tends to be worse without the pretraining stage, suggesting that the pretraining of ProtoCell4P on cells does benefit its performance on patient classification. The results also show that the model without cell type input can have comparable performance compared to the original version, which indicates the model can still learn how to perform patient classification from the scRNA-seq data without the knowledge of cell types. Nevertheless, though the performance of these two models is similar, the model without the knowledge of cell type is less explainable than the original ProtoCell4P model, because the high-level concepts of the key cells in the task are difficult to generalize without predefined knowledge such as cell types. Comparing the visualization results of cases from the model variants (see Fig. 5), we can see that different types of cells tend to be entangled with each other if the cell embedding module is not pretrained or the cell type information is not included, in which cases it will be difficult to summarize knowledge of cells for the classification task.

Table 6 also shows the performance of ProtoCell4P without subsampling is comparable to the default version on most tasks, indicating the model can manage to classify patients with only partial information on all cells, and that it is our design of the cell-centered classification architecture rather than data augmentation that allows the model to make precise predictions. However, the model without subsampling appears to perform better than the original model on the covid task, which is inconsistent with the results of other tasks. We attribute this to the fact that the average number of cells per sample in the covid dataset is much lower than in other datasets, which results in poorer quality of the subsamples when performing subsampling.

## 5 Conclusion

In this article, we identified the great potential of performing patient classifications based on single-cell data. Accordingly, we proposed an explainable prototype-based model, ProtoCell4P, to learn cell prototypes and collect prototype-related information from all cells for the classification. By including a cell embedding module, ProtoCell4P can leverage the knowledge of cells in scRNA-seq data to facilitate the patient classification tasks, alleviating the problem of sample scarcity. The design of an adaptive relevance scorer in the classification module helps the model to flexibly assign appropriate weights to different cells for more precise patient classification. Our approach is shown to be effective on all three classification tasks from real-world datasets and outperforms the existing methods in this area. Moreover, we demonstrated that the prediction result of our model can be interpreted by identifying the key cells for the patient’s classification and by summarizing the cell type knowledge on those discovered key cells. Our model can further uncover the association between cell types and the target phenotype classes of interest, which can be enlightening for related biological research.

## Data Availability

A cleaned version of the lupus dataset provided can be downloaded from https://github.com/yelabucsf/lupus_1M_cells_clean. The cardio dataset can be downloaded from https://singlecell.broadinstitute.org/single_cell/study/SCP1303. The covid dataset can be downloaded from https://singlecell.broadinstitute.org/single_cell/study/SCP1289.

## References

[btad493-B1] Ahn JH , Kim J, Hong SP et al Nasal ciliated cells are primary targets for SARS-CoV-2 replication in the early stage of COVID-19. J Clin Invest 2021;131:e148517. 10.1172/JCI148517 PMC8245175 34003804

[btad493-B2] Alvarez Melis D , Jaakkola T. Towards robust interpretability with self-explaining neural networks. In: *Proceedings of the 32nd International Conference on Neural Information Processing Systems* 2018, 7786–95.

[btad493-B3] Ballard DH. Modular learning in neural networks. In: *Proceedings of the sixth National Conference on artificial intelligence*, July 13–17, 1987, in Seattle, Washington, Vol. 647, 1987, 279–84.

[btad493-B4] Bien J , Tibshirani R. Prototype selection for interpretable classification. Ann Appl Stat 2011;5:2403–24.

[btad493-B5] Brbić M , Zitnik M, Wang S et al Mars: discovering novel cell types across heterogeneous single-cell experiments. Nat Methods 2020;17:1200–6. 33077966 10.1038/s41592-020-00979-3

[btad493-B6] Breda J , Zavolan M, van Nimwegen E. Bayesian inference of gene expression states from single-cell RNA-seq data. Nat Biotechnol 2021;39:1008–16. 33927416 10.1038/s41587-021-00875-x

[btad493-B7] Cao K , Brbic M, Leskovec J. Concept learners for few-shot learning. In: *9th International Conference on Learning Representations, ICLR 2021, Virtual Event, Austria*, *May 3–7, 2021.* OpenReview.net, 2021.

[btad493-B8] Chaffin M , Papangeli I, Simonson B et al Single-nucleus profiling of human dilated and hypertrophic cardiomyopathy. Nature 2022;608:174–80. 35732739 10.1038/s41586-022-04817-8 PMC12591363

[btad493-B9] Chua RL , Lukassen S, Trump S et al COVID-19 severity correlates with airway epithelium–immune cell interactions identified by single-cell analysis. Nat Biotechnol 2020;38:970–9. 32591762 10.1038/s41587-020-0602-4

[btad493-B10] Davis J , Goadrich M. The relationship between precision-recall and roc curves. In: *Proceedings of the 23rd International Conference on Machine Learning*, June 25-29, 2006, the campus of Carnegie Mellon University in Pittsburgh, Pennsylvania, 2006, 233–40.

[btad493-B11] Grønbech CH , Vording MF, Timshel PN et al ScVAE: variational auto-encoders for single-cell gene expression data. Bioinformatics 2020;36:4415–22. 32415966 10.1093/bioinformatics/btaa293

[btad493-B12] He B , Thomson M, Subramaniam M et al Cloudpred: Predicting patient phenotypes from single-cell RNA-seq. In: *Pacific Symposium on Biocomputing*, January 3-7, 2022 at the Fairmont Orchid on the Big Island of Hawaii, Hawaii, USA. World Scientific, 2021, 337–48. 34890161

[btad493-B13] Henriques A , Teixeira L, Inês L et al Nk cells dysfunction in systemic lupus erythematosus: relation to disease activity. Clin Rheumatol 2013;32:805–13. 23377197 10.1007/s10067-013-2176-8

[btad493-B14] Huai M , Liu J, Miao C et al Towards automating model explanations with certified robustness guarantees. In: *Proceedings of the AAAI Conference on Artificial Intelligence, virtual conference held February 22 – March 1, 2022*, Vol. 36, 2022, 6935–43.

[btad493-B15] Huang Y , Zhang P. Evaluation of machine learning approaches for cell-type identification from single-cell transcriptomics data. Brief Bioinform 2021;22:bbab035. 33611343 10.1093/bib/bbab035

[btad493-B16] Ianevski A , Giri AK, Aittokallio T. Fully-automated and ultra-fast cell-type identification using specific marker combinations from single-cell transcriptomic data. Nat Commun 2022;13:1246–10. 35273156 10.1038/s41467-022-28803-w PMC8913782

[btad493-B17] Johnson TS , Wang T, Huang Z et al Lambda: label ambiguous domain adaptation dataset integration reduces batch effects and improves subtype detection. Bioinformatics 2019;35:4696–706. 31038689 10.1093/bioinformatics/btz295 PMC6853662

[btad493-B18] Jovic D , Liang X, Zeng H et al Single-cell RNA sequencing technologies and applications: a brief overview. Clin Transl Med 2022;12:e694. 35352511 10.1002/ctm2.694 PMC8964935

[btad493-B19] Kim B , Rudin C, Shah JA. The Bayesian case model: a generative approach for case-based reasoning and prototype classification. In: *Proceedings of the 27th International Conference on Neural Information Processing Systems,* Vol. 2, 2014, 1952–60.

[btad493-B20] Kolodner JL. An introduction to case-based reasoning. Artif Intell Rev 1992;6:3–34.

[btad493-B21] Li O , Liu H, Chen C et al Deep learning for case-based reasoning through prototypes: a neural network that explains its predictions. In: Proceedings of the AAAI Conference on Artificial Intelligence, February 2–7, 2018 at the Hilton New Orleans Riverside, New Orleans, Louisiana, USA, Vol. 32, 2018.

[btad493-B22] Li X , Wang K, Lyu Y et al Deep learning enables accurate clustering with batch effect removal in single-cell RNA-seq analysis. Nat Commun 2020;11:2338–14. 32393754 10.1038/s41467-020-15851-3 PMC7214470

[btad493-B23] Lopez R , Regier J, Cole MB et al Deep generative modeling for single-cell transcriptomics. Nat Methods 2018;15:1053–8. 30504886 10.1038/s41592-018-0229-2 PMC6289068

[btad493-B24] Mandric I , Schwarz T, Majumdar A et al Optimized design of single-cell RNA sequencing experiments for cell-type-specific eQTL analysis. Nat Commun 2020;11:5504–9. 33127880 10.1038/s41467-020-19365-w PMC7599215

[btad493-B25] McInnes L , Healy J, Saul N et al UMAP: uniform manifold approximation and projection. JOSS 2018;3:861.

[btad493-B26] Perez RK , Gordon MG, Subramaniam M et al Single-cell RNA-seq reveals cell type–specific molecular and genetic associations to lupus. Science 2022;376:eabf1970. 35389781 10.1126/science.abf1970 PMC9297655

[btad493-B27] Petegrosso R , Li Z, Kuang R. Machine learning and statistical methods for clustering single-cell RNA-sequencing data. Brief Bioinform 2020;21:1209–23. 31243426 10.1093/bib/bbz063

[btad493-B28] Sibbitt W , Mathews P, Bankhurst A. Natural killer cell in systemic lupus erythematosus. defects in effector lytic activity and response to interferon and interferon inducers. J Clin Invest 1983;71:1230–9. 6853711 10.1172/JCI110872 PMC436983

[btad493-B29] Van der Maaten L , Hinton G. Visualizing data using t-SNE. J Mach Learn Res 2008;9:2579–605.

[btad493-B30] Wu M-L , Liu F-L, Sun J et al SARS-CoV-2-triggered mast cell rapid degranulation induces alveolar epithelial inflammation and lung injury. Signal Transduct Target Ther 2021;6:428. 34921131 10.1038/s41392-021-00849-0 PMC8677926

[btad493-B31] Xu Y , Zhang Z, You L et al scIGANs: single-cell RNA-seq imputation using generative adversarial networks. Nucleic Acids Res 2020;48:e85. 32588900 10.1093/nar/gkaa506 PMC7470961

[btad493-B32] Zhang Y , Lee T-Y. Revealing the immune heterogeneity between systemic lupus erythematosus and rheumatoid arthritis based on multi-omics data analysis. Int J Mol Sci 2022;23:5166. 35563556 10.3390/ijms23095166 PMC9101622

[btad493-B33] Ziegler CG , Miao VN, Owings AH et al Impaired local intrinsic immunity to SARS-CoV-2 infection in severe COVID-19. Cell 2021;184:4713–33.e22. 34352228 10.1016/j.cell.2021.07.023 PMC8299217

